# Inhibition of STAT3 with orally active JAK inhibitor, AZD1480, decreases tumor growth in Neuroblastoma and Pediatric Sarcomas *In vitro* and *In vivo*

**DOI:** 10.18632/oncotarget.930

**Published:** 2013-03-19

**Authors:** Shuang Yan, Zhijie Li, Carol J Thiele

**Affiliations:** ^1^ Cell & Molecular Biology Section, Pediatric Oncology Branch, Center for Cancer Research, National Cancer Institute

**Keywords:** neuroblastoma, Ewing sarcoma, rhabdomyosarcoma, JAK inhibitor, STAT3

## Abstract

The IL-6/JAK/STAT pathway is a key signal transduction pathway implicated in the pathogenesis of many human cancers, suggesting that kinase inhibitors targeting JAK/STAT3 may have a broad spectrum of antitumor activity. AZD1480, a pharmacological JAK1/2 inhibitor, exhibits anti-tumor potency in multiple adult malignancies. To evaluate the efficacy of inhibition of JAK/STAT3 signal transduction pathway we assessed the activity of AZD1480 in pediatric malignancies using preclinical models of three highly malignant pediatric solid tumors: neuroblastoma (NB), rhabdomyosarcoma (RMS) and the Ewing Sarcoma Family Tumors (ESFT). In this study, we employed panels of biomedical and biological experiments to evaluate the in vitro and in vivo activity of AZD1480 in NB, RMS and ESFT. Our data indicate that AZD1480 blocks endogenous as well as IL-6 induced STAT3 activation. AZD1480 decreases cell viability in 7/7NB, 7/7RMS and 2/2 ESFT cell lines (median EC_50_ is 1.5 μM, ranging from 0.36-5.37μM). AZD1480 induces cell growth inhibition and caspase-dependent apoptosis *in vitro* and decreases expression of STAT3 target genes, including cell cycle regulators CyclinD1, 3 and CDC25A, anti-apoptotic genes Bcl-2 and survivin, the metastasis-related factor TIMP-1 and c-Myc. *In vivo* studies showed AZD1480 significantly decreased tumor growth and prolonged overall survival in tumor-bearing mice. Tumors from AZD1480-treated mice showed inhibition of activated STAT3 as well as decreased expression of STAT3 downstream targets. Our study provides strong evidence of the anti-tumor growth potency of JAK inhibitor AZD1480 in pediatric solid tumors, providing proof-of principle that inhibition of the JAK/STAT3 signal transduction could be a promising therapeutic target for high-risk pediatric solid tumors.

## INTRODUCTION

Since the 1950s, the overall survival (OS) of pediatric cancer patients has increased from almost 0 to 80%. However, treatment of aggressive, high-risk neuroblastoma (NB), rhabdomyosarcoma (RMS) and Ewing sarcoma family tumors (ESFT) has remained a challenge. NB is the most common extra-cranial solid malignancy [1], RMS is the most common sarcoma and ESFT is the second most common bone tumor of childhood [1,2]. Children with localized, low- and intermediated-risk NB, RMS and ESFT are mostly curable, and have excellent long-term survival rates with standard therapies. In contrast, patients with high-risk aggressive NB, RMS and ESFT have a dismal outcome. Despite the current intensive therapy, the long-term event-free survival of high-risk NB is less than 40% [1,3], and the long-term OS in high-risk RMS and ESFT is only 30% and 39% [4,5,6,7], respectively. In addition, toxicity from current therapies is significant, leaving little room for further dose intensification. Therefore, new treatment strategies are urgently needed to improve the outcomes of patients with these malignancies.

Targeted therapy to mutant or dysregulated signal transduction pathway in human malignancies is a recent approach that has shown great promise when used alone or combined with conventional therapies. The Janus kinase (JAK) signal transducer and activator of transcription (STAT) pathway is one of them [8,9]. Activation of this pathway involves cytokine activation of its receptor, subsequent tyrosine phosphorylation of intracellular JAK kinases, then recruitment and phosphorylation of STAT transcription factors. Phosphorylated STAT proteins dimerize, translocate to the nucleus, and initiate target gene transcription. Cytokines of the interleukin-6 family, including IL-6, oncostatin M, leukemia inhibitory factor, are potent activators of JAK/STAT3 pathway, predominantly activating STAT3 through JAK1 and JAK2 [10]. Aberrant activation of JAK/STAT3 signaling, in particular STAT3, participates in the initiation, development and progression of human cancers via induction of STAT3 downstream genes that encode anti-apoptotic proteins, cell cycle regulators, and angiogenic factors such as Bcl-2, CyclinD1 and VEGF[11].

Aberrant activation of JAK/STAT3 signaling has been found in many adult and pediatric solid tumors. Increased STAT3 activity is frequently found in a wide variety of human tumors, including hematopoietic malignancies (leukemia, lymphoma, and multiple myeloma) as well as solid tumors (such as head and neck, breast, and prostate cancers) [12,13,14,15,16,17]. JAK2 mutations are not a major cause of activated JAK/STAT3 in pediatric solid tumors. Instead, elevated levels of IL-6 in the bone marrow and peripheral blood have been observed to be an independent marker of poor prognosis in high-risk NB patients [18]. *In vitro* studies demonstrated that bone marrow-derived IL-6 increased the proliferation and decreased the cytotoxic drug-induced apoptosis through activation of STAT3 in NB cells [19]. IL-6 has not been directly studied in the pathogenesis of RMS or ESFT. However, increased macrophage infiltration and tumor microvascular density have been noted in tumors from ESFT patients with poor prognoses [20]. Since tumor-associated macrophages express higher concentrations of cytokines including IL-6 [20], increased IL-6 may be one mechanism that leads to aberrant activation of JAK/STAT3 pathway in pediatric sarcomas. In addition, activation of JAK/STAT3 pathway may be maintained by its induction of SIPR1, which has been shown to generate an autocrine positive feedback loop in many solid tumor cells and a paracrine feedback loop with cells in their microenvironment [21]. Furthermore, elevated levels of activated STAT3 are found in ESFT and RMS tumor tissues as well as cell lines [22,23]. These findings suggest that the aberrant activation of JAK/STAT3 pathway participates in the pathogenesis of pediatric solid tumors and targeting key components of this pathway may represent a promising strategy to treat these malignancies.

To test whether inhibition of the JAK/STAT3 pathway would affect the growth of pediatric solid tumors, we evaluated the anti-tumor activity of AZD1480, an ATP competitive inhibitor of JAK1 and JAK2, which has been shown to decrease the growth of adult tumors in several pre-clinical models [24,25,26,27,28]. In this study, we found that AZD1480-mediated inhibition of the JAK/STAT3 pathway resulted in *in vitro* and *in vivo* suppression of tumor growth in neuroblastoma, rhabdomyosarcoma and Ewing sarcoma. As a proof of concept this demonstrates that blockade of the JAK/STAT3 signaling may have therapeutic benefit for pediatric patients with these solid malignancies.

## RESULTS

### AZD1480 treatment inhibited the growth of pediatric solid tumor cell lines *in vitro*

**Figure 1:**
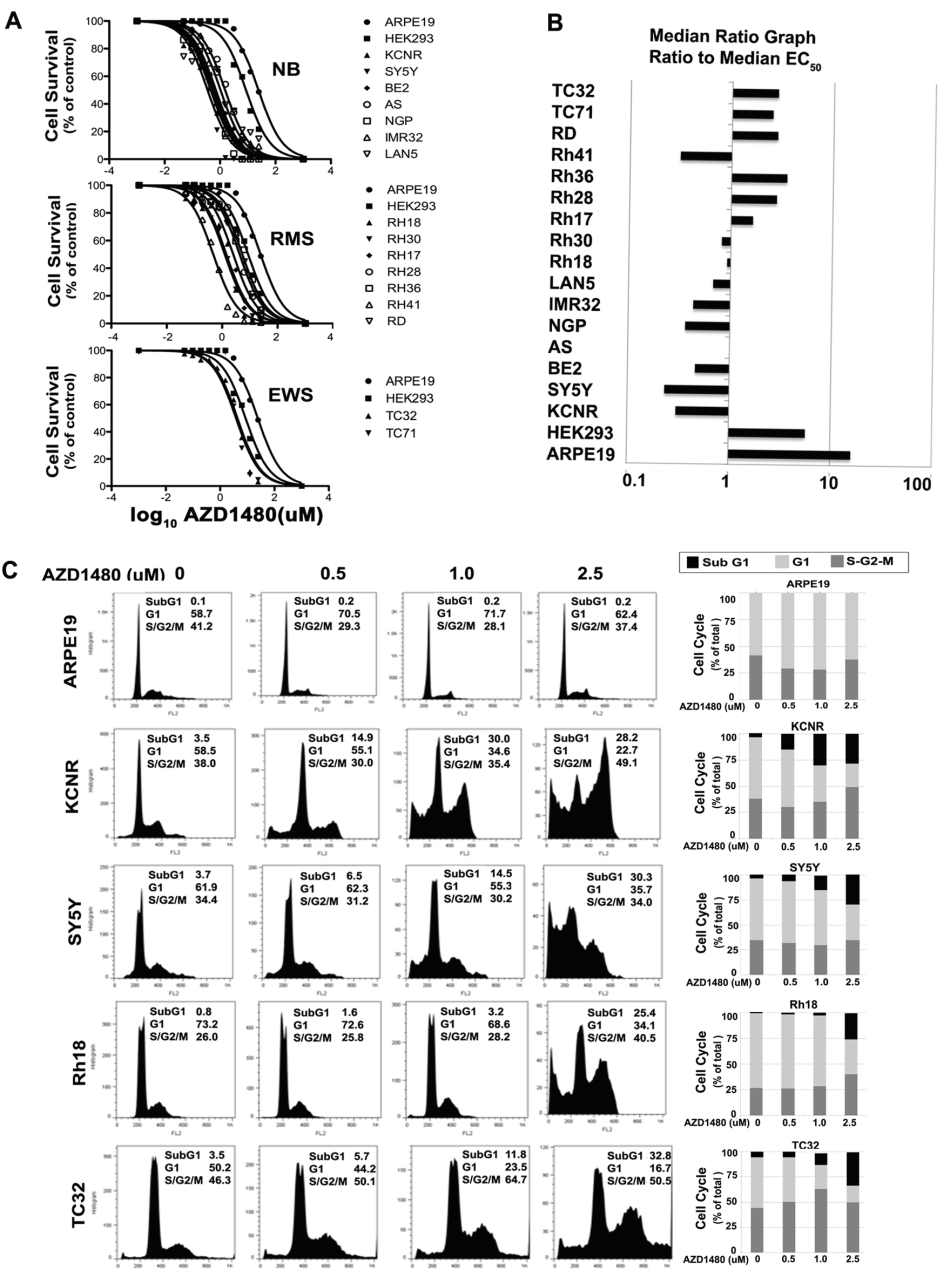
The effects of AZD1480 on high-risk pediatric tumor cell lines *in vitro* A) Typical growth inhibition curves determined by MTS assays for the NB, RMS and ESFT tumor cell lines compared with normal control ARPE19 and HEK293 cell lines. B) The median EC_50_ ratio graph shows the relative EC_50_ values for all the cell lines tested. Each bar represents the ratio of the panel EC_50_ to the EC_50_ value of the indicated cell line. Bars to the right represent cell lines with less sensitivity, while bars to the left indicate cell lines with higher sensitivity to AZD1480. C) Effect of AZD1480 on the cell cycle of pediatric solid tumors. Two NB cell lines (KCNR and SY5Y), one RMS cell line (Rh18), one ESFT cell line TC32 and the normal ARPE19 cell line were treated with various concentrations of AZD1480 (0, 0.5, 1 and 2.5 μM) for 72 hours. Cells were stained with propidium iodide and analyzed by flow cytometer. Results are tabulated within the graph and graphically shown in histograms (left). The bars represent the percentages of cells different phases of the cell cycle; sub-G1 (black), G1 (light grey) and the S + G2/M phase (dark grey).

AZD1480 activity was evaluated by MTS assay in 7 NB, 7 RMS, and 2 ESTF tumor cell lines and 2 immortal but non-tumorigenic cell lines, ARPE19 and HEK293T. After 72 hours, all AZD1480-treated cell lines displayed a dose-dependent decrease in cell number (Fig. 1A). The median EC50 *in vitro* was 1.5 μM. There was a 69-fold range in EC_50_ values, with the most sensitive cell line being the NB cell line SY5Y with an EC_50_ of 0.36 μM. The immortalized normal cell line ARPE19 was the least sensitive with an EC_50_ of 24.4 μM. As Figure 1B and Table 1 showed, 5/7 NB and 1/7 RMS cell lines were relatively more sensitive to AZD1480 with the Panel EC_50_/Median EC_50_ less than 0.5; 2/7 NB and 3/7 RMS showed median sensitivity to AZD1480 (0.5< Panel EC_50_/Median EC_50_ <1.5); 2/2 ESFT and 3/7 RMS were less sensitive (1.5< Panel EC_50_/Median EC_50_ <5). The 2 non-tumorigenic cell lines ARPE19 and HEK293T were the least sensitive (Panel EC_50_/Median EC_50_ >5). This shows that the tumor cell lines were more sensitive to AZD1480-mediated inhibition of cell proliferation than the normal cells. Four cell lines were selected for further *in vitro* and *in vivo* analyses: SY5Y (single copy-MYCN) and KCNR (MYCN-amplified) from NB which were in group that was most sensitive to AZD1480; Rh18 (RMS) which was in the group showing intermediate sensitivity to AZD1480 and TC32 (ESFT) which was in the group showing the least sensitivity to AZD1480.

**TABLE 1:** In vitro Activity of AZD1480

Cell line	Histotype	Relative EC50(uM)	Panel R- EC50 cell line R-EC50
ARPE19	Non-tumorigenic	24.38	15.97
HEK293	Non-tumorigenic	8.67	5.67
KCNR	Neuroblastoma	0.46	0.30
SY5Y	Neuroblastoma	0.36	0.23
BE2	Neuroblastoma	0.71	0.46
AS	Neuroblastoma	1.53	1.00
NGP	Neuroblastoma	0.56	0.37
IMR32	Neuroblastoma	0.66	0.43
LAN5	Neuroblastoma	1.04	0.68
RH18	Rhabdomyosarcoma	1.42	0.93
RH30	Rhabdomyosarcoma	1.25	0.82
RH17	Rhabdomyosarcoma	2.51	1.65
RH28	Rhabdomyosarcoma	4.28	2.80
RH36	Rhabdomyosarcoma	5.37	3.52
RH41	Rhabdomyosarcoma	0.48	0.31
RD	Rhabdomyosarcoma	4.32	2.83
TC32	Ewing sarcoma	3.85	2.52
TC71	Ewing sarcoma	4.33	2.84
Median		1.53	1.00
Minimum		0.36	0.23
Maximum		24.38	15.97

To determine the events that led to the AZD1480-induced decrease in cell proliferation, alterations in cell cycle were analyzed by flow cytometry in cells treated with AZD1480 for 72 hours. As shown in Figure 1C, there was an increase in cells in the subG1 and G2/M phases of the cell cycle with the increasing dose of AZD1480 (0 to 2.5 μM). AZD1480 treatment had little to no effect on cell cycle distribution of the non-tumorigenic ARPE19 cell line at these concentrations.

**Figure 2:**
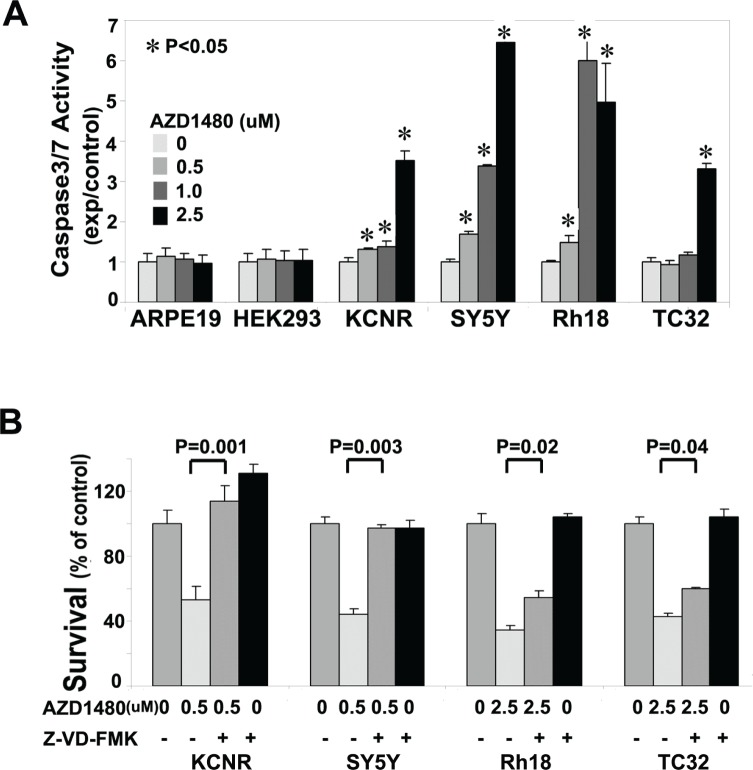
AZD1480 induced caspase3/7-dependent apoptosis A) Induction of caspase-3/7 activity by AZD1480 in pediatric NB and sarcoma cell lines. Tumor cell lines (KCNR, SY5Y, Rh18 and TC32) and normal cell lines (ARPE19 and HEK293) were treated with different concentrations of AZD1480 (0, 0.5, 1.0 and 2.5 μM) for 24 hours. Caspase-3/7 activity was determined using a Caspase-Glo3/7 Assay Kit. A representative graph of 3 independent experiments is depicted. Data represent mean±SD, of triplicates within 1 experiment (*t*-test). *, P<0.05 for AZD1480-treated cells vs. vehicle-control cells. B) Rescue of AZD1480-induced cell growth arrest by Z-VAD-FMK. KCNR, SY5Y, Rh18 and TC32 cells were pretreated with Z-VAD-FMK (50 μM) for 3 hours, followed by AZD1480-treatment (0.5 μM for KCNR and SY5Y, 2.5 μM for Rh18 and TC32) for additional 72 hours, or treated with AZD1480 or Z-VAD-FMK alone for 72 hours. MTS assay was used to assess cell survival. Data represent mean±SD of triplicate samples from a representative experiment of 3 independent experiments performed. P values were indicated for AZD1480/ Z-VAD-FMK-co-treated cells vs. AZD1480-treated cells (*t*-test).

To assess whether the AZD1480-induced cell death was mediated via a caspase-dependent pathway, we performed a caspase-3/7 activity assay. AZD1480-treated pediatric tumor cell lines showed a significant increase in caspase-3/7 activity in all the tumor cell lines tested (Fig. 2A). AZD1480 induced an increase in caspase 3/7 activity in KCNR, SY5Y and Rh18 at the concentration of 0.5 μM. However, caspase 3/7 activity did not change in the TC32 cells until the AZD1480 concentration reached 2.5 μM. In the two non-tumorigenic cell lines, AZD1480, even at 2.5uM, failed to induce a significant change in Caspase3/7 activity. This indicated AZD1480 had a specific effect on tumor cells. To assess whether the activation of Caspase 3/7 was critical for AZD1480-induced cell death, cells were treated with pan-caspase inhibitor Z-VAD-FMK prior to AZD1480-treatment (Fig. 2B). The pan-caspase inhibitor Z-VAD-FMK blocked, to differing extents, the cytotoxic activity of AZD1480 in all 4 tumor cell lines. Compared to the AZD1480-treated group, Z-VAD-FMK treatment significantly rescued survival (Fig. 2B). These data indicate that AZD1480 induces caspase-dependent cell death in these 4 pediatric solid tumor cell lines.

### AZD1480 inhibited both endogenous constitutive and IL-6-induced STAT3 activation in pediatric cells

**Figure 3:**
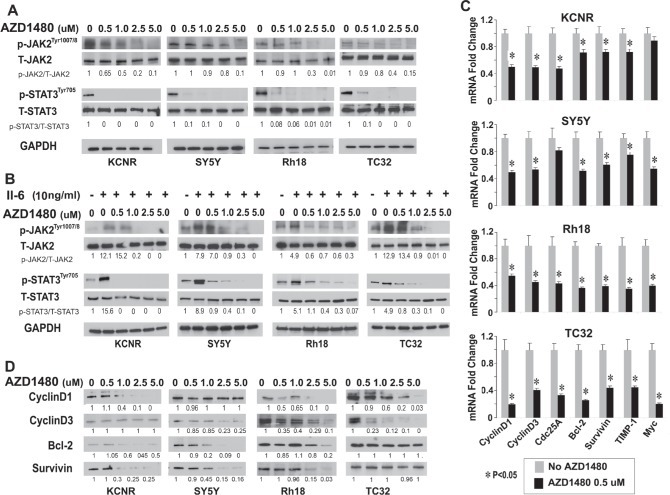
Inhibition of JAK/STAT3 signaling by AZD1480 in pediatric cells *in vitro* A and B) AZD1480 blocks endogenous constitutive (A) and IL-6-induced (B) activation of JAK2 and STAT3. Cells were treated with indicated doses of AZD1480 alone for 24 hours (A), or pretreated with IL-6 (10 ng/ml) for 15 minutes, washed with PBS 3 times and then followed by treatment with indicated doses of AZD1480 for 24 hours (B), then lysed and immunoblotted with indicated antibodies. Ratios of p-STAT3/T-STAT3 and p-JAK2/T-JAK2 shown under the representative blots were normalized to that of untreated-control (normalized as “1”) in each cell line. C and D) AZD1480 decreased STAT3-downstream target gene expression. Cells were treated with indicated doses of AZD1480 for 24 hours. C. Real-time PCR was performed for indicated STAT3 target genes. Data represent mean±SD, of 3 independent experiments. *, P<0.05 was indicated for AZD1480-treated cells vs. control cells (*t*-test). D. Immunoblots were performed as described in Materials and Methods for indicated STAT3-downstream targets. Ratios of the detected target/GAPDH shown under the representative blots was normalized to that of untreated-control (normalized as “1”) in each cell line.

As an ATP competitive inhibitor of JAK1 and JAK2, AZD1480 was recently shown to inhibit activation of STAT3 and depress the growth of multiple adult tumors [25,26]. AZD1480 (0, 0.5, 1, 2.5 μM) treatment inhibited the constitutive levels of activated JAK2 and activated STAT3 without changing the total protein levels of JAK2 and STAT3 (Fig 3A). Since studies indicated that bone marrow-derived IL-6 increased the proliferation and decreased the cytotoxic drug-induced apoptosis through activation of STAT3 in NB cells [19], we evaluated whether AZD1480 would affect this signal transduction pathway. As shown in supplementary Figure 1A, IL-6Rα/gp80 protein was detected in 8/8 and gp130 protein expression was detected in 7/8 cell lines. IL-6 was detected in the conditioned medium of 4/8 cell lines (Supplementary Fig. 1B). AZD1480 inhibited the IL-6-induced activation of JAK/STAT3 signaling *in vitro* (Figure 3B). To determine whether inhibition of STAT3 phosphorylation affected STAT3 target gene expression, we analyzed the expression of selected STAT3 direct target genes (CyclinD1, CyclinD3, Cdc25a, Bcl-2, survivin, TIMP-1 and c-Myc) by qPCR and immunoblots. After 24 hours of AZD1480-treatment, there was a significant decrease in the mRNA levels of 6/7 STAT3 target genes in KCNR and SY5Y, and 7/7 STAT3 in Rh18 and TC32 (Fig. 3C). The protein levels of selected STAT3 targets decreased, albeit to variable levels (Fig. 3D). We also detected a significant decrease in the levels of secreted VEGF in 7/8 tumor cell lines tested (Supplementary Fig 2). AZD1480 also inhibited the migration ability of KCNR and TC32 cells but not of SY5Y and Rh18 cells using a wound closure assay (Supplementary Fig 3). These data indicates that consistent with the decreased STAT3 activity, AZD1480 repressed the expression of STAT3 target genes involved in cell-cycle regulation (CyclinD1, CyclinD3, Cdc25a), apoptosis (Bcl-2, survivin and c-Myc) as well as genes implicated in migration and invasion (TIMP-1 and VEGF) in pediatric solid tumor cells.

### AZD1480 inhibited tumor growth *in vivo* and prolonged the survival of tumor-bearing mice

**Figure 4:**
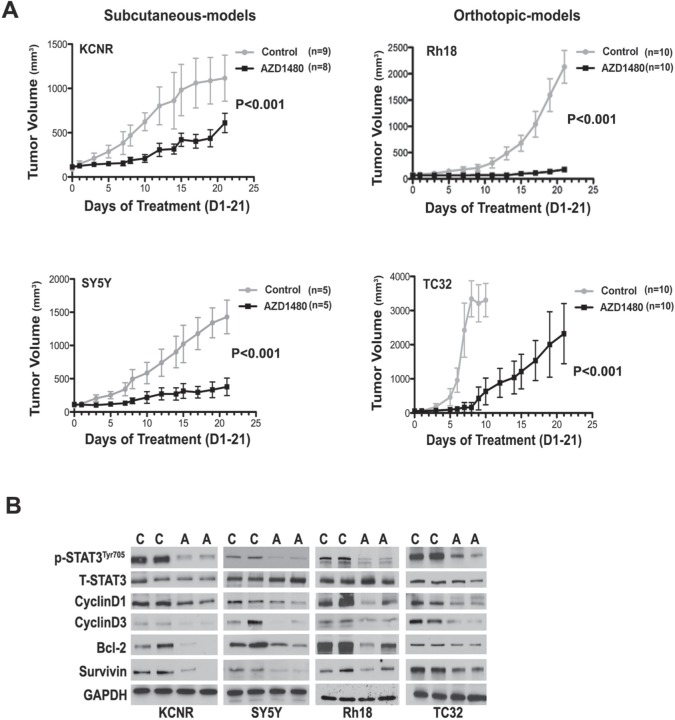
AZD1480 inhibited STAT3 activity and growth of NB, RMS and ESFT xenografts *in vivo* Subcutaneous xenografts of NB were established by injection of 2×10^6^ of NB cells (KCNR and SY5Y) into the right flank of 4-6 week-old female nude mice. Orthotopic xenografts of RMS and ESFT were established by injecting 2×10^7^ Rh18 or TC32 cells into the left gastrocnemius muscle of SCID/Beige mice. AZD1480 treatment were initiated when tumors reaches the size at 100-200 mm^3^. AZD1480 and vehicle were administered daily for up to 3 weeks (30 mg/kg QD for KCNR and SY5Y, 30 mg/kg BID for Rh18 and TC32) by oral garage. Tumor sizes were measured three times a week. A) The graph represents a comparison of mean tumor volumes between control and AZD1480-treated groups in each cell line during the course of AZD1480 or placebo treatment. Data represent mean ± SD. P value between control and AZD1480-treated group in each cell line was determined by a two-way ANOVA. B) Effect of AZD1480 on STAT3 phosphorylation and its downstream targets in tumor xenografts *in vivo*. Two mice in each group (C=Control group, A=AZD1480-treated group) were killed after 9 doses of AZD1480-treatment in KCNR, SY5Y, Rh18 and TC32, respectively. Tumors were excised and proteins were extracted. Total proteins (15 μg) were analyzed for phosphorylated (p) -STAT3 ^Tyr705^, total (T)-STAT3, CyclinD1, CyclinD3, Bcl-2, Survivin and GAPDH by immunoblotting.

To determine the effect of AZD1480 on tumor growth *in vivo*, we employed heterotypic subcutaneous NB xenograft (KCNR and SY5Y) and orthotopic RMS (Rh18) and ESFT (TC32) xenograft models. As shown in Figure 4A and Supplementary Figure 4, tumor growth in AZD1480-treated group was significantly depressed compared to control in each cell line (P< 0.001 using a two-way ANOVA). To evaluate the effect of AZD1480 on STAT3 activation *in vivo*, we collected tumor samples from mice after 9-doses of AZD1480 or vehicle. Western blot analyses revealed that tumors from mice treated with AZD1480 had decreased levels of tyrosine phosphorylated STAT3 as well as of STAT3 downstream targets (CyclinD1,-3, Bcl-2 and Survivin) compared to the levels in tumors from mice receiving vehicle (Fig, 4B). This shows that AZD1480 treatment induces the inhibition of STAT3 activity and its target gene expression *in vivo*.

**Figure 5:**
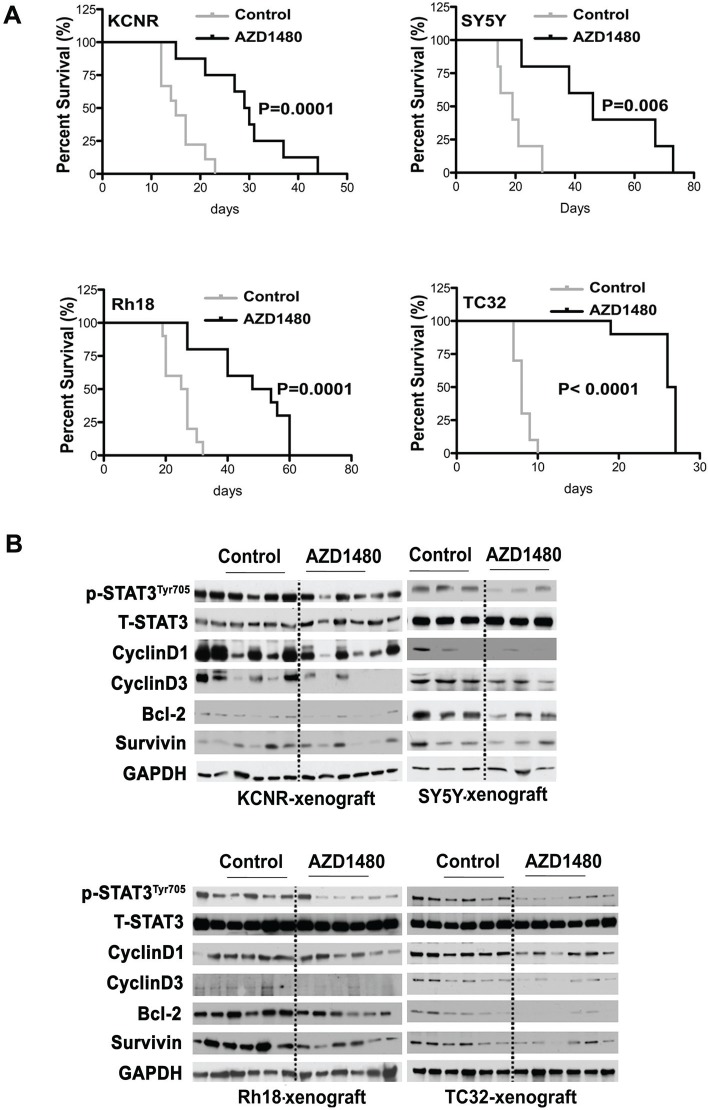
Beneficial effect of AZD1480 on the survival of tumor-bearing mice To evaluate survival in the cohort of mice treated in Fig. 4, the treatments were halted at 3 weeks and tumor xenograft growth was monitored in mice. When tumor xenografts reached a maximal diameter of 2 cm, the mice were euthanized. A) Survival curve was plotted by Kaplan-Meier analysis. P value was calculated using a two-sided long-rank test. B) After mice were sacrificed, tumor samples were frozen for immunoblotting to evaluate the late inhibition effect of AZD1480 on STAT3 activation and its downstream targets.

After AZD1480 treatments were stopped, mice were euthanized when tumor growth reached a diameter of 2 cm. Kaplan-Meier survival curves from the commencement of AZD1480 treatment until mice were euthanized indicated that there was a significant survival advantage for the AZD1480-treated mice in groups bearing KCNR (P=0.006), SY5Y (P=0.001), Rh18 (P=0.001) and TC32 (P=0.001) tumors compared with mice that had received the vehicle control (Fig 5A). The median survival date was markedly increased for mice in the AZD1480-treated cohort vs. vehicle control in all tumor models evaluated: KCNR (29.5 vs. 15 days); SY5Y (46 vs. 19 days); Rh18 (51 vs. 26 days) and TC32 (26.5 vs. 8 days). These data indicated that AZD1480 treatment significantly reduced the tumor burden and prolonged the survival of tumor-bearing mice in the NB xenografts (KCNR and SY5Y) grown in a heterotypic site as well as the RMS (Rh18) and ESFT (TC32) xenografts grown in orthotopic sites.

**Figure 6:**
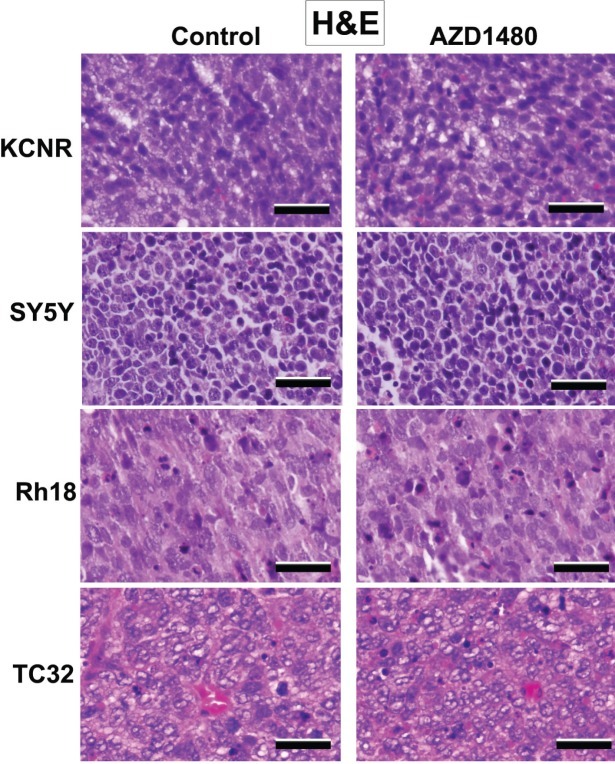
Histological evaluation by hematoxylin-eosin (HE) staining on SY5Y, KCNR, Rh18 and TC32 xenografts Tumor tissues from euthanized mice were fixed in formalin and sectioned for histological evaluation by HE staining. The magnification of the representative pictures is ×20 (scale bar =100 μm).

Western blot analyses of proteins taken from tumors obtain at time of euthanasia were used to evaluate changes in gene expression (Fig. 5B). We observed a decrease in several STAT-3 targets such as, CyclinD1, cyclinD3, Bcl-2 in the tumors treated with AZD1480. The H & E staining of representative tumor xenografts (Fig. 6) and the images in Supplementary Figure 5 showed that the tumors express human-HLA antigens indicating the cells in the xenografts were of human origin.

## DISCUSSION

Management of high-risk NB, ESFT and RMS remains a challenge for pediatric oncologists. Effective, targeted therapies with differing toxicity profiles from cytotoxic drugs are needed. Dysregulation of the JAK2/STAT3 pathway has been noted in a number of pediatric solid tumors. We found the JAK1/2 inhibitor AZD1480 inhibited cell proliferation via induction of G2/M cell cycle arrest and Caspase3/7-dependent apoptosis. Moreover, AZD1480 suppressed the growth of NB, RMS and ESFT xenografts *in vivo*. AZD1480 blocked endogenous constitutive and cytokine-induced activation of STAT3 *in vitro* and inhibited the activation of STAT3 in tumor xenografts. This was associated with decreased expression of STAT3 downstream target genes such as Bcl-2, CyclinD1 and Survivin *in vitro* and *in vivo*.

Currently, therapeutic blockade of IL-6/JAK/STAT3 signaling using IL-6 ligand-binding antibodies, IL-6R blocking antibodies, or specific compounds that inhibit the activity of JAK and STAT3 have been tested pre-clinically for prostate cancer, non-small cell lung cancer, ovarian cancer, breast cancer and colon cancer. Based on these promising studies, some approaches are in early clinical trials for the treatment of adult solid tumors (IL-6 mAb S0354 (NCT00433446), STAT3 inhibitors OPB-31121 (NCT00955812), OPB-51602 (NCT01423903), and JAK1/2 inhibitor AZD1480 (NCT01112397) which was recently closed). However, none of these approaches has been systematically evaluated in pediatric solid tumors. Herein we provide evidence of the anti-tumor effect of blocking JAK2/STAT3 pathway by using the JAK2 inhibitor AZD1480 in 3 different types of pediatric solid tumor models.

The growth of the NB cell lines was particularly sensitive to the anti-tumor activity of AZD1480 *in vitro* with 7/7 cell lines at or below the median EC50. The pediatric sarcoma cells were relatively less sensitive to AZD1480 *in vitro* with 4/7 RMS cell lines (RD, Rh17, Rh28 and Rh36), and 2/2 EWS cell lines (TC32 and TC71) with EC50 values greater than the median EC50. However, the growth of the orthotopic RMS and ESFT tumor xenografts were significantly depressed by AZD1480. This may be due to the fact that the daily dose of AZD1480 was doubled (compared to the NB xenografts). It is also possible that AZD1480 mediated anti-angiogenic activity [31,32] may contribute to the anti-tumor effect in the orthotopic *in vivo* models. In all cases it should be noted that the mice were only treated for a total of 21 doses (as specified by AstraZeneca) and a longer duration of AZD1480 treatment may have resulted in an even greater increase in survival of the mice.

Mechanistically, we showed that AZD1480 inhibits the JAK/STAT3 pathway as measured by suppression of several specific, well-established downstream transcriptional targets of STAT3 in cells *in vitro* and in tumor xenografts *in vivo*. The decreases in STAT3 phosphorylation and its downstream targets such as Bcl-2 and CyclinD1 and D3 detected in this study are consistent with findings in previous studies in adult tumors which found that AZD1480 induces decreases in STAT3 targets [25,26,28]. Recently, AZD1480 has been reported to have anti-angiogenic and anti-metastatic activity via inhibition of VEGF and MMP9 [31,32]. In our study the levels of TIMP-1 mRNA, a factor associated with invasiveness and secreted VEGF, a known pro-angiogenic factor, were decreased after treatment with AZD1480. Thus the expression of a variety of downstream targets of activated STAT3 was inhibited by AZD1480. While AZD1480 treatment inhibited STAT3 targets at the transcriptional level, the decreased mRNA levels did not always result in similar or consistent decreases in the protein levels of the various STAT3 targets. These inconsistencies between mRNA and protein levels of STAT3 targets may be due to differences in post-transcriptional regulation of these proteins in the different cell lines tested. It is also possible that the inability to decrease protein levels of Bcl-2 and survivin which occurred in the TC32 and Rh18 cell lines may be related to their decreased sensitivity to AZD1480 *in vitro*.

In this study we evaluated AZD1480 as a single agent. The *in vivo* responses to AZD1480 were limited to tumor growth inhibition, as no objective responses (tumor regressions) were observed for either subcutaneous or orthotopic xenografts in our study. Activated STAT3 signaling has been reported to be a marker predictive of drug resistance [33]. Inhibition of STAT3 activity enhances chemosensitivity of multiple tumor types to a number of different cytotoxic agents or other targeted agents [33,34,35]. Therefore, in our future studies, we will evaluate agents directly targeting STAT3 alone and in combination with chemotherapy or targeted-therapies such as AKT and mTOR inhibitors.

In summary we provide the first pre-clinical proof of concept of the anti-tumor potency of inhibition of JAK/STAT3 pathway in pediatric solid tumors utilizing AZD1480. This study indicates that blockade of JAK/STAT3 signal transduction pathway may be a promising therapeutic target in high-risk pediatric solid tumors.

## MATERIALS AND METHODS

### Cell lines and Reagents

Human NB cell lines (KCNR, BE2, SY5Y, AS, NGP, LAN5 and IMR32), EWS cell lines (TC32 and TC71) and RMS cell lines (Rh17, Rh18, Rh28, Rh30, Rh36, Rh41 and RD) were maintained as previously described [29,30] and determined to be genetically pure using a single-nucleotide polymorphism-based genotype assay (kindly performed by SJ Chanock, Division of Cancer Genetics and Epidemiology, NCI). Human embryonic kidney cell line HEK293 and human retinal pigment epithelial cell line ARPE19 were obtained from ATCC (Manassas, VA).

AZD1480, a JAK1/2 inhibitor, was synthesized and provided by AstraZeneca (Waltham, MA). For *in vitro* studies, AZD1480 was dissolved as a 20mM stock solution in DMSO and frozen in aliquots at -80°C. For *in vivo* experiments, AZD1480 was suspended in water supplemented with 0.5% hypromellose and 0.1% Tween 80 (20mg/ml), stored at 4° C and freshly made every week. Human IL-6 was purchased from Miltenyi Biotec (Auburn, CA). Antibodies against phosphorylated STAT3 (Y705), STAT3, CyclinD1, CyclinD3, Bcl-2 and Survivin were purchased from Cell Signaling Technologies (Beverly, MA). The GAPDH antibody was purchased from Santa Cruz (Santa Cruz, CA).

### *In vitro* Analysis of Cell viability and Cell Cycle

Cells were plated in 96-well plates (3000-5000 cells per well) in triplicate, incubated overnight and then treated with AZD1480 (0.39 to 25μM), or vehicle DMSO for 72 hours. Where indicated a pan caspase inhibitor, Z-VAD-FMK (50 μM) (R&D Systems, MN) was added to cells 3 hours before AZD1480 treatment. Parallel plates were prepared for cell viability assays using of the 3-(4, 5-dimethylthiazol-2-yl)-5-(3-carboxymethoxyphenyl)-2-(4-sulfophenyl)-2H-tetrazolium, inner salt assay (MTS) assay (Promega, WI) as previously described (34). The absorbance (490nm) was detected using a Versamax microplate reader (Molecular Devices, CA). Cell viability was normalized to untreated cells. The concentration of half-maximal effective inhibition of viability (EC50) was determined by using Prism 4.0 software (GraphPad Software Inc, CA). Each experiment was done in triplicate and results were averaged.

Cell cycle analyses were as described previously (34). Cells treated with AZD1480 or vehicle control, DMSO, for 72 hours were harvested, washed twice with PBS and stained with propidium iodide (Sigma- Aldrich Corp, MO) for DNA content determination. Flow cytometric data were acquired using a Cytek modified FACScan flow cytometer (Becton Dickinson, CA) and analyzed using FlowJo Software (Tree Star, OR). Experiments were performed in triplicate.

### Assay of Caspase3/7 Activity *in vitro*

Cells were seeded into 96-well plates at a density of 1 × 10^4^ cells per well, in triplicate, cultured overnight and then treated with 0, 0.5, 1 and 2.5 μM AZD1480 for another 24 hours. The combined activity of caspase-3/7 was evaluated using the Caspase-Glo 3/7 Assay Kit (Promega, WI) according to the manufacturer's instruction [29]. Experiments were performed in triplicate.

### Real-Time PCR and Protein Analyses

Total RNA was extracted using RNeasy Mini Kit (Qiagen, CA) and reverse-transcribed to cDNA with SuperScript III First-Strand Synthesis SuperMix (Invitrogen, CA). The levels of mRNA expression of STAT3 target gene CyclinD1, CyclinD3, CDC25A, BCL-2, Survivin, TIMP-1 and c-Myc in cells treated with AZD1480 or vehicle were evaluated by quantitative real-time PCR (qPCR) using an ABI Prism 7000 (Applied Biosystems, CA) with SYBR Green SuperMix according to the manufacturer's protocol. β-Actin was used for input normalization. Validated primers used for detection were obtained from RealTimePrimers.com.

Lysates of total protein were isolated using Qproteome Mammalian Protein Prep Kit (Qiagen, CA) and concentrations were measured using a BCA Protein Assay Kit (Pierce, IL). Proteins (15 μg) were separated by SDS-PAGE gels and then transferred to nitrocellulose membranes. Membranes were blocked by 5% non-fat milk in TBST (0.01% Tween) for 1 hour, incubated with primary antibodies overnight at 4° C, followed by 1 hour incubation with HRP-conjugated secondary antibodies and then developed with Western Lighting-ECL (Perkin Elmer, NY). Densitometric analysis of appropriately exposed autoradiographs was performed using NIH Image 1.63 software. Relative protein levels (phosphorylated STAT3/ total STAT3, phosphorylated JAK2/ total JAK2, STAT3 downstream targets/ GAPDH) were calculated from quantified data. Ratios shown under the representative blots were normalized to that of untreated-control (normalized as “1”) in each cell line. All qPCRs and immunoblots were performed in triplicate.

### AZD1480 treatment of xenograft tumors

For NB xenograft model, 5-6-week-old female athymic nude mice (Taconic, NY) were injected subcutaneously with 2 × 10^6^ cells (KCNR and SY5Y). For RMS and ESFT xenograft models, SCID/Beige mice (4–6 weeks old; Charles River Laboratories, MA) were orthotopically injected with 2 × 10^6^ cells (TC32 or Rh18) per mouse into the left gastrocnemius muscle. When the subcutaneous tumors reached 100-200 mm^3^ or the minimum diameter of tumor in orthotropic mice reached 0.5 cm, mice bearing KCNR, SY5Y, TC32 or Rh18 were randomly assigned into an AZD1480 treatment group or a control group. The AZD1480 group received a once daily oral gavage of AZD1480 (30 mg/kg QD per mouse for KCNR and SY5Y, 30 mg/kg BID per mouse for Rh18 and TC32) or vehicle alone respectively for 21 days. To detect the effect of AZD1480 on tumor growth, tumor size was measured three times a week using calipers and calculated as previously described: the equation for subcutaneous-xenografts in KCNR and SY5Y is (*L* ×*W*^2^)/4, where *L* = length (millimeter) and *W* = width (millimeter); and for orthotopic-xenografts of Rh18 and TC32 is (*D* × *d*^2^)/6 × 3.12, where *D* is the maximum diameter and *d* is the minimum diameter, respectively [29,30]. To evaluate the effect of AZD1480 on its *in vivo* targets, two mice in each group were randomly selected and sacrificed on day 9 after initiation of treatment and the tumor tissue was frozen for analysis of protein levels of activated-STAT3 and its downstream targets by western blot analyses. These mice were not included in either tumor growth or animal survival analyses.

To determine the effect of AZD1480 on survival of tumor-bearing mice, we counted the days from the initiation of treatment (AZD1480 or control) to the time the tumors reached a diameter of 2 cm (end point as required by NIH Animal Care and Use Committee). Tumor tissue isolated at the time the mouse was euthanized was either snap frozen and stored at −80°C for protein analysis by western blot analyses, or fixed in 10% formalin, sectioned, and stained with hematoxylin–eosin (American Histo Labs, MD) or anti-human HLA antibodies by immunohistochemistry. All xenograft studies were approved by the Animal Care and Use Committee of the National Cancer Institute in accordance with the institutional guidelines (PB–023).

### Evaluation and Statistical Analysis

Statistical analyses were performed with the GraphPad Prism software. Statistical significance was established at P< 0.05. Kaplan Meier survival curve comparisons were performed using a two-way ANOVA.

## Supplementary Figures and Methods




