# Supplementary material for: Inhibition of STAT3 with orally active JAK inhibitor, AZD1480, decreases tumor growth in Neuroblastoma and Pediatric Sarcomas In vitro and In vivo
Source: Oncotarget. 2013 Mar 19;4(3):433–45. doi: 10.18632/oncotarget.930 (PMC3717306; doi:10.18632/oncotarget.930)
Supplement: Supplementary file 1 [file oncotarget-04-433-s001.pdf]

Inhibition of STAT3 with orally active JAK inhibitor, AZD1480, decreases tumor growth in Neuroblastoma and Pediatric Sarcomas *In vitro* and *In vivo* - *Yan et al*

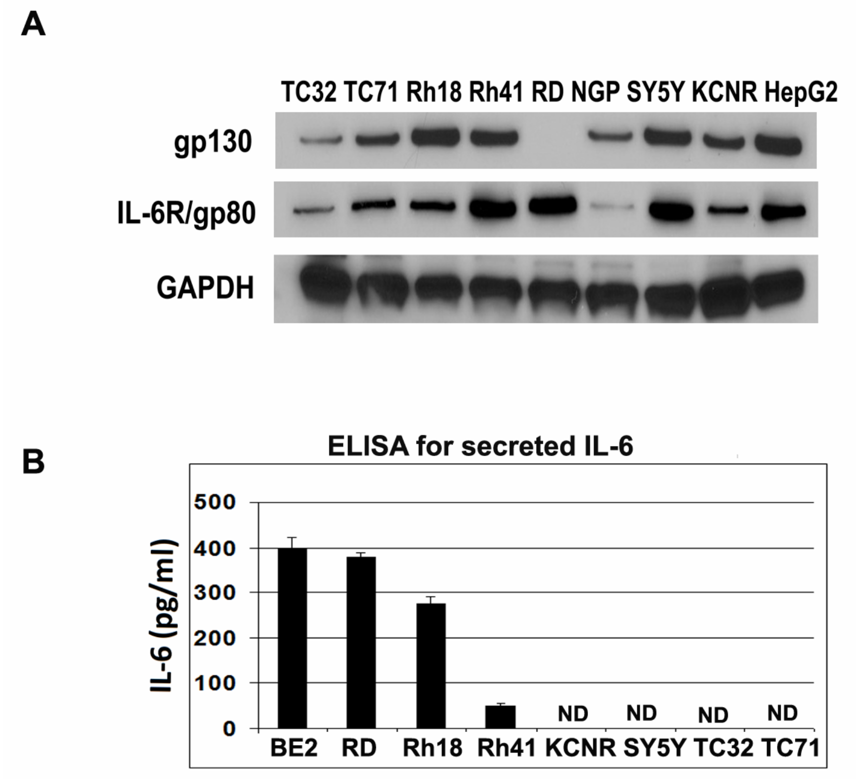

Supplementary Figure 1: IL-6R/gp80, gp130 expressions in pediatric solid tumor cell lines and IL-6 secretion. (A) Western blot analysis demonstrated the presence of the ubiquitous gp130 receptor protein and IL-6Rα/gp80 protein in 8/8 pediatric solid tumor cell lines tested. (B) IL-6 was detected in unconcentrated serum-free media of 4 cell lines (BE2, RD, Rh18 and Rh41) but not in the KCNR, SY5Y, TC32, TC71 cell lines whose levels were below the level of detection of the assay (0.1 pg/ml). ND, not detectable (below 0.1 pg/ml).

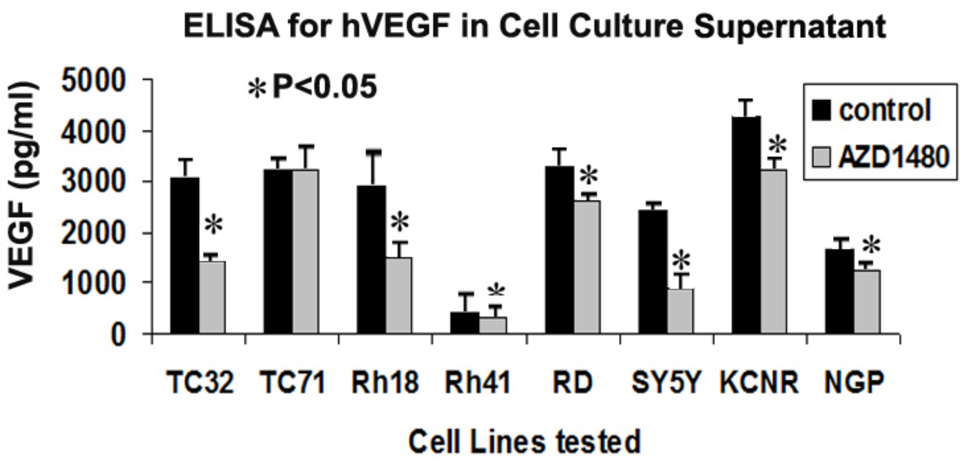

Supplementary Figure 2: AZD1480 decreased the secretion of VEGF in pediatric solid tumor cells. Levels of human VEGF in the medium of cultured cells treated with or without AZD1480 (0.5μM, 16 hours) were determined by ELISA. AZD1480 significantly decreased the secretion of VEGF in 7/8 of the cell lines evaluated ($P<0.05$, *t*-test).

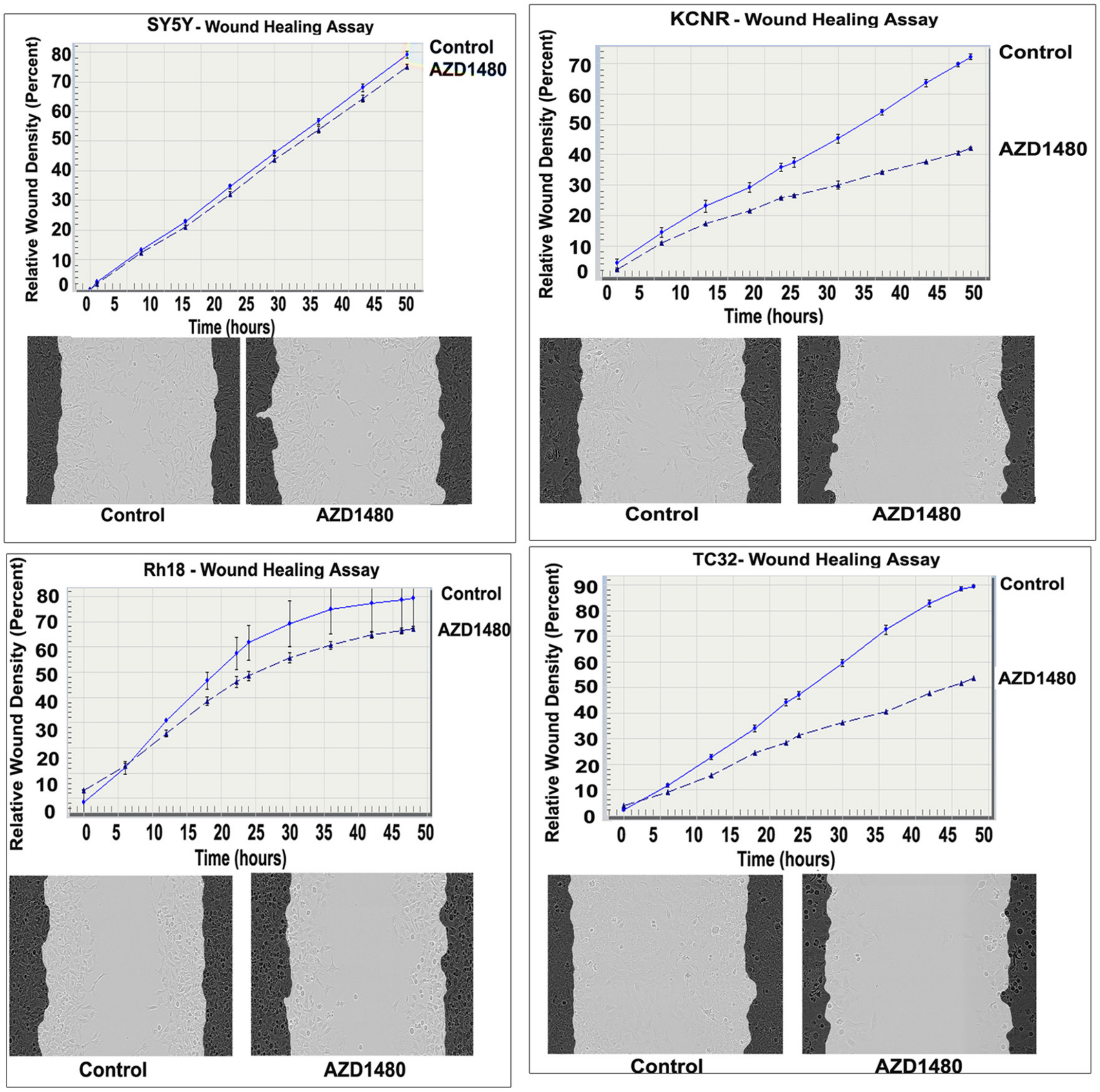

Supplementary Figure 3: AZD1480 repressed the migration ability of KCNR, and TC32 *in vitro.* Wound confluence was graphed over time to quantitatively evaluate the characteristics of wound closing. The IncuCyte Scratch Assay Software was used to analyze the data. AZD1480-treatment caused a significant reduction in the migration of KCNR, and TC32 cells compared with the migration of the untreated control cells ($P<0.05$, Two-way ANOVA). AZD1480 had no effect on migration of SY5Y or Rh18 cells ($P>0.05$, Two-way ANOVA). A representative phase-contrast photomicrograph of cells at the end point of the experiment was shown at the bottom for each cell line tested.

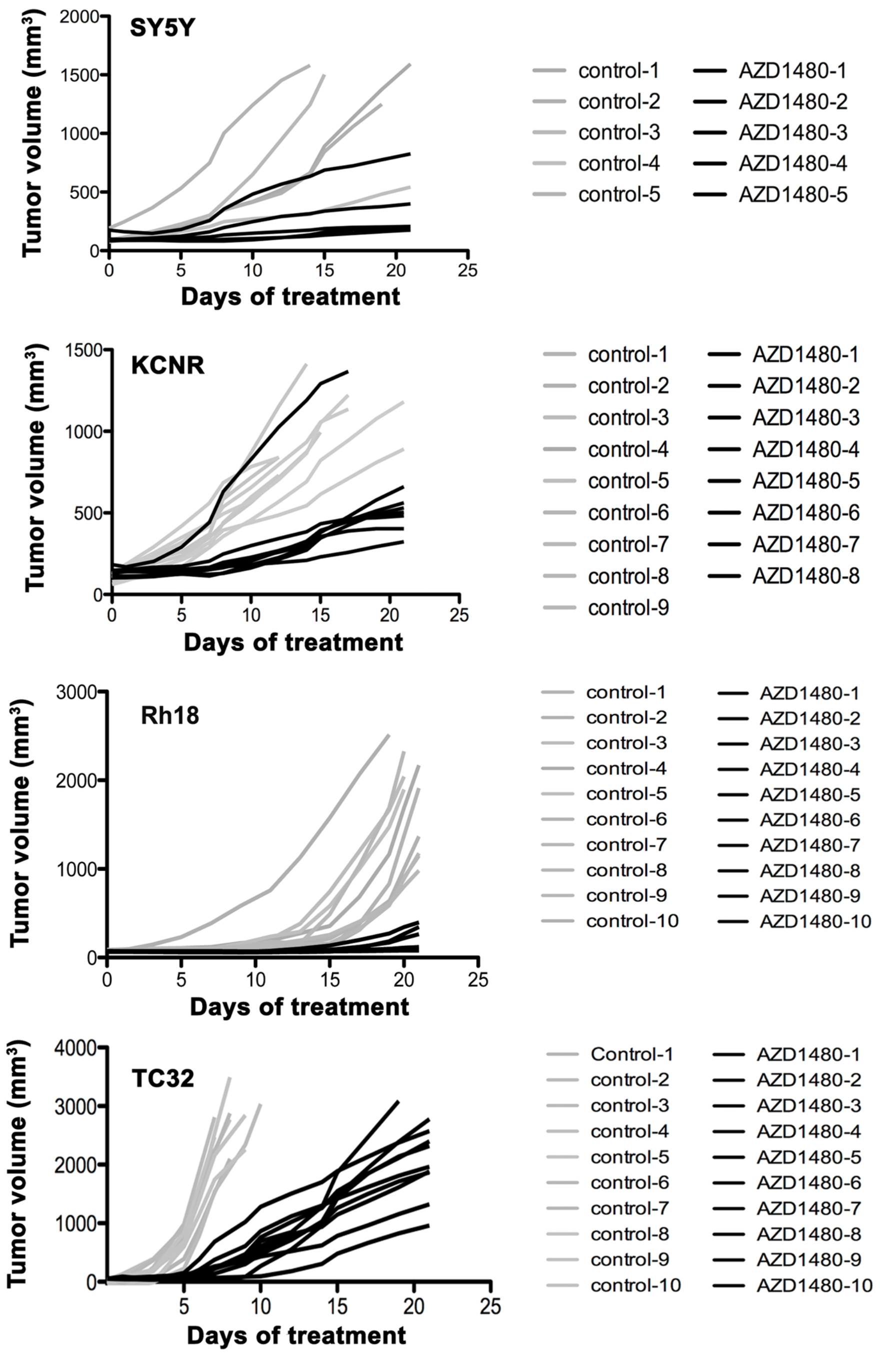

Supplementary Figure 4: Individual tumor growth curve *in vivo.* Effect of AZD1480 on tumor growth in vivo was established as described in Figure 4. AZD1480 and vehicle placebo were administrated orally for 3 weeks. The tumor sizes were measured 3 times a week. The tumor growth from each mouse was plotted during the time period of AZD1480 treatment (Day 1 to Day 21). Each cell line has a plotted graph indicating the control and AZD1480-treated mice.

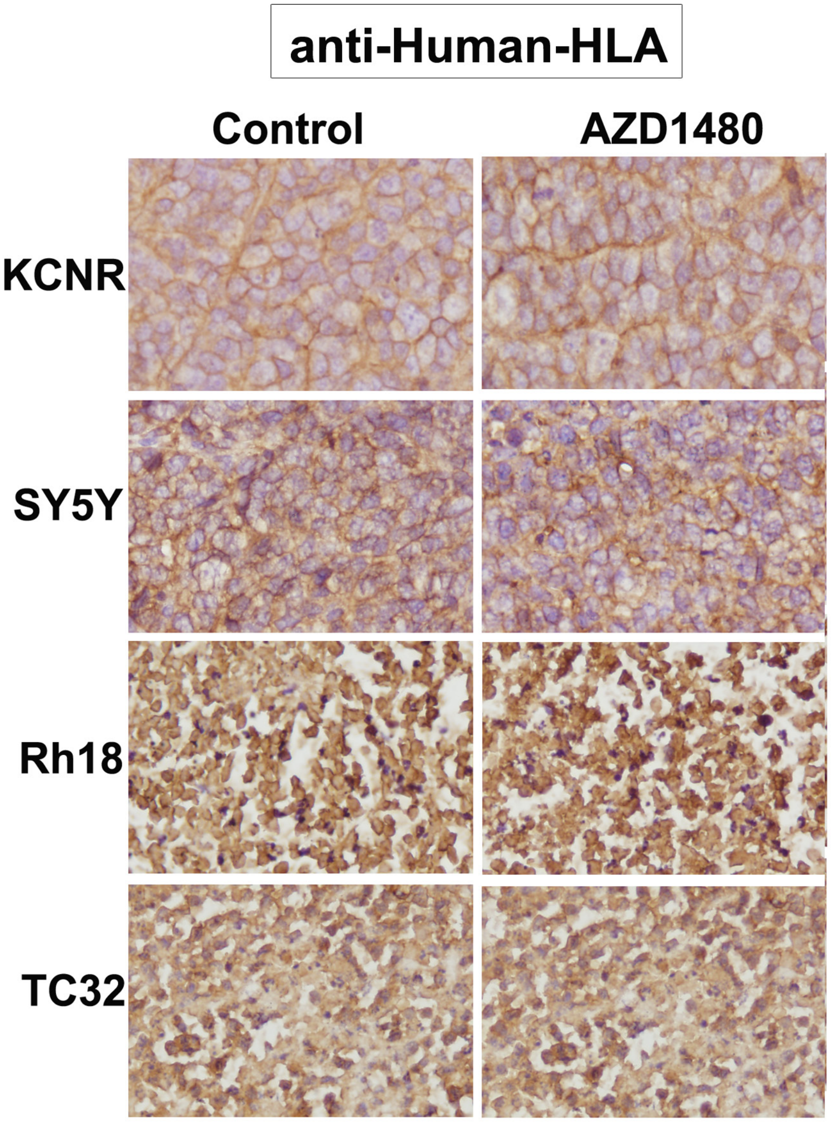

Supplementary Figure 5: Immunohistochemical staining for human-HLA in the xenograft tumor tissue. When the maximal diameter of tumor reached 2 cm, mice were killed and tumor samples were sectioned for IHC staining to evaluate the tumor histology.
