# Supplementary material for: Inhibition of STAT3 with orally active JAK inhibitor, AZD1480, decreases tumor growth in Neuroblastoma and Pediatric Sarcomas In vitro and In vivo
Source: Oncotarget. 2013 Mar 19;4(3):433–45. doi: 10.18632/oncotarget.930 (PMC3717306; doi:10.18632/oncotarget.930)
Supplement: Supplementary file 2 [file oncotarget-04-433-s002.pdf]

Material and Method for Supplementary Data

Western blot analysis for IL-6R/gp80 and gp130 expression and ELISA assay for IL-6 secretion

The expression levels of IL-6R/gp80 and gp130 in pediatric cell lines were determined by Western Blot analysis. Antibodies were purchased from R&D system (Anti-human IL-6R antibody, cat# MAB227; Anti-human gp130 antibody, cat# MAB2281). HepG2, a human liver hepatocellular carcinoma cell lines, was used for positive control for the expression of IL-6R/gp80 and gp130.

Levels of human IL-6 in the medium of cultured cells (KCNR, SY5Y, BE2, RD, Rh18 Rh41, TC32, and TC71) were determined in triplicate by Enzyme Linked Immunoabsorbance Assay (ELISA) according to the manufacturer's protocol (Human IL-6 ELISA Kit from Thermo Scientific).

ELISA assay for secreted VEGF levels

Levels of human VEGF in the medium of cultured cells treated with or without AZD1480 (0.5uM, 16 hours) were determined in triplicate by Enzyme Linked Immunoabsorbance Assay (ELISA) according to the manufacturer's protocol (Quantikine Human VEGF Kit, R&D Systems).

Wound-healing assays *in vitro*

KCNR, SY5Y, Rh18 and TC32 cells were plated into Essen ImageLock 24-well plates, incubated overnight and treated with or without AZD1480 (0.5 µM) for 16 hours. The medium was changed to complete medium with mitomycin-C (10 µg/ml) for 2 hours to inhibit cell proliferation. After washing with low serum medium (0.5% FBS) three times, the confluent monolayer of cells was scratched using the Essen 24-well Wound Maker (Ann Arbor, MI, USA) following the manufacture's protocol. The cells were cultured in low serum medium (0.5% FBS) with or without AZD1480 (0.1 µM). The wound confluence was obtained and analyzed using the IncuCyte phase-contrast imaging and scratch wound assay system (ESSEN INSTRUMENTS, MI).

Individual tumor growth curve *in vivo*

Effect of AZD1480 on tumor growth *in vivo* was established as described in Figure 4 and Materials and Method section. AZD1480 and placebo were administrated orally for 3 weeks. The tumor sizes were measured 3 times a week. The tumor growth of each mouse was plotted during the time of treatment (Day 1 to Day 21). Each cell line has a plotted graph indicating the growth of each mouse in vehicle or AZD1480-treated group.

Immunohistochemistry of Xenograft Tumor Tissue

The tumor tissue was fixed in 10% formalin, sectioned (5 µm-thick sections) for immunohistochemical staining. Sections were deparaffinized with xylene, 100% ethanol, and 95% ethanol. Antigen retrieval was performed with Tris/EDTA buffer with pH at 9.0. The sections were incubated with 3% hydrogen peroxide to quench endogenous peroxidase activity, followed by incubation with 5% goat serum to block nonspecific

protein binding and incubation overnight with mouse-anti-human-HLA-DR (5 µg/ml, ab20181, Abcam). Sections were subsequently labeled using biotinylated goat-anti-mouse secondary IgG antibody and streptavidin-conjugated horseradish peroxidase (Dako). The sections were counter-stained with hematoxylin, washed, and mounted with mounting medium (Dako). Images were obtained in the National Institutes of Health core imaging facility.
